# Fungal inoculation of human fecal-woodchip substrates produces species-specific effects on indicator bacteria and substrate degradation

**DOI:** 10.1093/sumbio/qvag043

**Published:** 2026-10-05

**Authors:** Jules Peter, Adriana Clavijo Daza, Elizabeth Tilley

**Affiliations:** Global Health Engineering, Institute of Science, Technology and Policy, Department of Mechanical and Process Engineering (D-MAVT), ETH Zurich, 8092 Zurich, Switzerland; Global Health Engineering, Institute of Science, Technology and Policy, Department of Mechanical and Process Engineering (D-MAVT), ETH Zurich, 8092 Zurich, Switzerland; Global Health Engineering, Institute of Science, Technology and Policy, Department of Mechanical and Process Engineering (D-MAVT), ETH Zurich, 8092 Zurich, Switzerland

**Keywords:** fungal sanitation, composting toilet, fungal fecal treatment, myco-sanitation

## Abstract

Safely treating human feces in decentralized, waterless toilets remains a sanitation challenge. Filamentous fungi degrade lignocellulose and stay active under the dry conditions that limit bacterial degradation, yet their potential to treat human feces has rarely been quantified. In this exploratory screening study, we inoculated four filamentous fungal species represented by five isolates (two *Pleurotus ostreatus* isolates and one isolate each of *Sordaria fimicola, Trichoderma harzianum*, and *Ganoderma lucidum*) onto fresh human feces mixed with beechwood chips and followed colonization, mold contamination, pH, fecal indicator bacteria (*Escherichia coli* and *Enterococcus* spp.), and dry weight over 14 days. Species differed markedly. *Sordaria fimicola* colonized every sample and fully suppressed competing molds but raised bacterial concentrations and pH. *Ganoderma lucidum* and both *P. ostreatus* isolates kept the substrate acidic and suppressed fecal odor; where it established, *G. lucidum* reduced both indicator bacteria below detection, though its colonization was slow and unreliable. *Trichoderma harzianum* grew fast but died back after 8 days. Fungal colonization curbed spontaneous molds and for *S. fimicola* and *T. harzianum* accelerated substrate degradation, revealing species-specific trade-offs between reliable colonization, suppression of fecal indicator bacteria, and pH control, and pointing to promising candidates and mechanisms for biological fecal treatment.

**Sustainability statement**

This study contributes to Sustainable Development Goal 6 (Clean Water and Sanitation), particularly target 6.2 on adequate and equitable sanitation. Safely treating human feces in decentralized, waterless toilets remains a barrier to protecting health and enabling nutrient reuse. By quantifying how filamentous fungi colonize fresh fecal-woodchip substrates and alter fecal indicator bacteria, pH, and substrate degradation, this exploratory screening identifies candidate fungi and mechanisms for biological fecal treatment. Developing this approach could improve pathogen inactivation and fertilizer quality in dry toilets, delivering safer sanitation.

## Introduction

Decentralized sanitation is vital in areas where sewered sanitation is absent. To meet this need, various decentralized technologies have been developed to treat feces and that, additionally, allow the reuse of human waste as fertilizer. Such technologies require effective pathogen inactivation and odor control; must produce high-quality and safe end products that allow for reuse and must be acceptable by users. Low-cost decentralized solutions include composting toilets and urine diversion dry toilets (UDDTs). Both types of toilets are waterless, making them good solutions for regions where water is scarce. Contrary to urine, which is mostly safe to reuse, fecal material contains high concentrations of pathogens that require treatment to be safe.

In UDDTs, pathogen inactivation relies on desiccation, requiring storage for up to 1–2 years. Additives such as ash, lime, or sawdust are used to accelerate drying and suppress fecal odor (Magri *et al*. 2013). Pathogen inactivation is strongly dependent on environmental conditions and the additives used. Rewetting of the substrate causes pathogen reactivation and odor; resistant helminth eggs such as *Ascaris lumbricoides* can survive even extended storage times (Magri *et al*. 2013). Treatment based on drying, not degradation, results in a final product that resembles dried fecal material and wood chips or ash rather than compost (Tilley *et al*. 2014). Poor nitrogen mineralization produces inferior fertilizer quality with delayed nitrogen availability (Mnkeni and Austin 2009).

To increase fertilizer quality and reduce possible risks, a hot composting step would be beneficial. However, thermophilic composting relies on the addition of organic waste that is often difficult to obtain in needed volumes at household systems (Vinnerås *et al*. 2003). Furthermore, composting systems are challenged by unbalanced C:N ratios, anaerobic pockets within the substrate, dry edges, and wet bottoms (Anand and Apul 2014). Temperatures that guarantee pathogen inactivation are difficult to reach and maintain within these systems, yielding an unsafe substrate (Redlinger *et al*. 2001, Tønner-Klank *et al*. 2007, Anand and Apul 2014). The carbon-to-nitrogen ratio of human feces of 8:1 can easily be adapted with carbon-rich additives to an optimal 30:1 (Anand and Apul 2014, Rose *et al*. 2015). However, organic material dries out the fecal mass, halting bacterial degradation, which requires high water activity. Furthermore, at ambient temperatures, most bacteria struggle to degrade recalcitrant substrates, such as wood or straw, which are often used as additives (Richard *et al*. 2002, Ahn *et al*. 2008). And although bacterial additives are commercially available for pit latrines, there is no proof that they have a significant effect on substrate degradation or bacterial numbers (Bakare *et al*. 2015). The reasons for this are the already high bacterial concentration in the substrate outcompeting the added bacteria and the added bacteria not being adapted to the harsh conditions in pit latrines (Bakare *et al*. 2015, Saxena and Den 2021). Notably, these additives target wet systems such as pit latrines and septic tanks, whereas in the much drier fecal lignocellulose substrate of dry toilets, bacterial degradation will halt.

Contrary to bacteria, filamentous fungi can tolerate lower water activity and secrete enzymes that degrade recalcitrant lignocellulose under non-thermophilic conditions. It is well documented that filamentous fungi play a crucial role in dung degradation in natural ecosystems (Richardson and Watling 1968, Calaça *et al*. 2024). Although human feces are nitrogen-rich (5%–7%) and fiber-poor compared to herbivore dung, mixing feces with lignocellulose, such as straw or woodchips, creates a composition suitable for fungal growth (Rose et al. 2015).

Fungi can help eradicate pathogenic bacteria through direct predation, secretion of antimicrobial compounds, or competition for nutrients (Künzler 2018, Hamad *et al*. 2022, Solomon *et al*. 2025). Fungal enzymes are effective degraders of pharmaceutical residues, polyaromatic hydrocarbons, and other chemical contaminants (Torres-Farradá *et al*. 2024). Fungal pre-treatment optimizes animal manure composting by shortening stabilization periods and increasing temperature and product quality (Varma *et al*. 2015, Huhe 2017, Meng et al. 2019). Despite these potential benefits, fungal treatment of human feces in decentralized sanitation systems has rarely been investigated. Preliminary projects using a *Pleurotus ostreatus* mycelium lining in diversion toilets suppressed the fecal smell and suggested an effect on fecal indicator bacteria (Urieli 2016, Dahmen *et al*. n.d.). However, no rigorous data has been published.

This study investigated the growth potential of filamentous fungi in fresh, untreated human fecal material with high indicator bacteria concentrations, a high nitrogen content, and competing molds. Four species (five isolates) were tested: two strains of *P. ostreatus*, the species used by Urieli (2016) and Dahmen *et al*. (n.d.); *Sordaria fimicola* (isolated from human feces); *Trichoderma harzianum* T22 (selected for biocontrol activity); and *Ganoderma lucidum* (for fast growth). To simulate UDDT conditions, fresh feces were mixed in a 2:1 ratio with wood chips. Colonization success, competing mold incidence, pH changes, *Escherichia coli* and *Enterococcus* spp. concentrations, and dry weight loss were evaluated after 14 days.

## Materials and methods

### Fungal strains

The strains *P. ostreatus* MG1015 and *P. ostreatus* MG1010, and *G. lucidum* MG11500 were procured commercially from Mycogenetics (Germany). The dung-inhabiting fungus *S. fimicola* was isolated from human fecal samples by introducing mycelium onto malt extract agar (MEA) plates. *Trichoderma harzianum* T22 was received from the fungal collection of the Extension Arable Crops group from Agroscope, Switzerland.

### Storage and inoculum preparation

To sustain fungal cultures throughout the experimental period, a circle with a 5 mm diameter was stamped out of the old plate and transferred to the middle of a new MEA plate maintained at 25°C for 2 weeks before being used in the assay.

### Fecal material collection

Fecal samples from a single individual were used consistently throughout the experiment. The samples were collected using a fecal catcher (Abbexa, Cambridge, UK), transferred into a 3-l freezing bag (Freshstar Clic-Lock, Galaxus), stored in an airtight container (Rothobox 3 l, Galaxus), and immediately refrigerated at 5°C. The collected fecal samples were stored for a maximum of 1 day at 5°C before use. A single donor was used to isolate the effect of the inoculated fungi from inter-individual variation in gut microbiome, diet, and substrate physicochemistry; fecal sample was included as a random intercept in all models to account for differences between the six samples from the same donor. The study was conducted in accordance with the Declaration of Helsinki. Ethical approval was granted by the ETH Zurich Ethics Commission (project 25 ETHICS-119). The fecal donor provided written informed consent to participate. No identifiable personal information is presented.

### Substrate preparation

Pure fecal samples were weighed and homogenized by kneading in an aseptic freezing bag for ~2 min. Beechwood chips (“Chipsi”, fine beech wood chips, Galaxus) were added to the bag at a 2:1 (w/w) ratio. The mixture was then homogenized again by manual kneading for two minutes. Of the mixture, 15 ± 1 g was transferred to petri dishes (LLG 90 × 16 mm, with vents) and spread out using a sterile plastic spatula.

### Inoculation

Plates were inoculated immediately after preparation using 5 mm agar plugs from fungal-colonized MEA plates that were ~14–16 days old. An individual fungal plate was used for each replicate.

### Incubation

Inoculated and control plates were incubated at 25°C and ~80% relative humidity for 14 days. Plates were randomly positioned within the incubator and reshuffled after each sampling timepoint to minimize positional effects. Control treatments consisted of uninoculated fecal-woodchip substrate prepared identically to treatment plates.

### Growth measurements

Plates were photographed using a mobile phone camera (iPhone 11, Apple) on a pole at a fixed distance of 13.5 cm. To identify each picture, a QR code was placed next to the plate to identify the fungal species, treatment ID, and inoculum ID. Plates were photographed at multiple timepoints between 1 and 14 dpi. Imaging days varied by fecal batch: each batch was imaged on eight to ten of days 1, 2, 3, 5, 6, 7, 8, 9, 10, 12, 13, and 14 dpi. All batches had the common timepoints (2, 6, 7, 8, and 14 dpi). To measure the mycelial area, the images were analyzed using ImageJ (V.1.54p, http://imagej.org) by a single operator manually drawing lines around the growth areas. The scale was set to 221 pixels per centimeter. Colony growth was quantified as the projected (two-dimensional) colony area, a non-destructive metric recorded repeatedly on the same plate at each imaging timepoint and used as a proxy for colonization extent. The visual fungal contamination was measured in the same manner. Where inoculated fungal growth and contaminant growth overlapped, the overlapping area was added to both measurements.

### pH measurements

A pH meter (LLG-ph meter 5, LLG Labware) was used to measure the pH of the diluted sample. The pH value was measured at 0 dpi after sample preparation using a sample directly from the homogenized fecal sample bag. Samples at 14 dpi were collected from three random points within the mycelial growth zone and combined into a single sample of 1 ± 0.1 g, which was suspended in 2 ml of distilled water for pH measurement.

### *Escherichia coli* concentration measurements

For the 0-dpi time point, three samples were taken randomly from the homogenized mixed fecal substrate. For the 14 dpi timepoint, samples of ~1 g were taken from three random positions within the mycelium zone. Samples were weighed and filled into a 15 ml centrifuge tube (LLG-Zentrifugenröhrchen economy, PP 15 ml, LLG-Labware). Depending on the expected bacterial counts, 1 or 5 ml 0.85% (w/v) NaCl solution (0.145 M) was added to the sample. Each tube was shaken for 1 min using a vortex (VORTEX-GENIE 2, Scientific Industries, Inc.). Further dilutions, if needed, were done using 0.85% NaCl (0.145 M) in reaction tubes (2 ml Reaktionsgefäss, BRAND GMBH + CO). A volume of 100 µl of the diluted sample was streaked onto agar plates. *Escherichia coli* was enumerated using TBX chromogenic agar (Carl-Roth, Karlsruhe, Germany). *Enterococcus* spp. were enumerated using *Enterococcus*-selective agar (Merck, Darmstadt, Germany). The plates were incubated overnight at 37°C. Colonies were counted the next day for *E. coli* and after 2 days for *Enterococcus*. After streaking, the samples were immediately placed back in the refrigerator. If too many or too few colonies grew on the plates, then the streaking was repeated with an adapted dilution the next day for *E. coli* or 2 days for *Enterococcus*. The limit of detection (LOD) varied depending on the initial dilution factor and sample weight; at the lowest dilution [1 g sample in 1 ml 0.85% NaCl (0.145 M), 100 µl plated], the LOD was ~10 colony-forming units (CFU)/g.

### Dry weight change

At the start of the experiment, the weight of each empty petri dish was recorded, and the total weight of the plates with fecal material was recorded after filling the plates. The water content was used to calculate the dry weight of each plate. To calculate the dry weight change, the initial substrate water content was measured by taking three samples of ~5 g randomly of the mixed substrate. For the treatments, around 5 g was sampled from three random spots over the whole plate. Samples were weighed onto pre-weighed aluminum foil pieces, and both weights were recorded. The samples were dried in a drying oven at 105°C overnight. Dry weight was calculated according to Equation 1:


(1) \begin{eqnarray*}
{\mathrm{dry\ weight}} &=& \left( {{\mathrm{total\ wet\ weight\ }} - {\mathrm{\ plate\ weight}}} \right)\\&& {\mathrm{*}}\left( {\frac{{{\mathrm{sample\ dry\ weight\ }} - {\mathrm{\ foil\ weight}}}}{{{\mathrm{sample\ wet\ weight\ }} - {\mathrm{\ foil\ weight}}}}} \right).
\end{eqnarray*}

For the initial dry weight, the mean of three random samples of the initial mixture of ~5 g were used.

### Data analysis

Data analysis was performed using R (version 4.5.2) within the RStudio environment (v2024.04.1). Two thresholds were established to categorize the fungal growth. The 90 mm plates provided ~63.6 cm² of substrate surface; the ≥20 cm² and ≥54 cm² thresholds therefore correspond to roughly 30% and 85% of the available plate area, representing coverage sufficient for representative sampling and near-complete colonization, respectively. First, a threshold of >20 cm² was set to define “initial growth”, ensuring that substrate samples for pH, bacterial concentrations, and dry weight were collected from areas with confirmed mycelial coverage rather than from uncolonized or contamination-dominated substrate. Plates below this threshold were excluded from physiological analyses because sampling would not reflect the effect of the inoculated fungus. Lacking fungal inoculation and requiring no filtering, all control replicates were included in the physiological comparisons. Because this filtering removes proportionally more low-success plates (controls, *G. lucidum, P. ostreatus*), overall growth success rates including all plates are reported separately. Consequently, this filtered subset was used to assess the growth speed, contamination resistance, pH, bacterial concentrations, and dry weight loss. Conversely, the complete unfiltered dataset was used to analyze the overall growth success and contamination rates. Finally, “successful colonization” was defined as at least 54 cm² of colonized area combined with a mycelium health score of ≥3 at 14 days post-inoculation (dpi), indicating that the species was dominant and alive on the substrate. The mycelium health score was assessed on a 0–5 scale by a single unblinded operator: 0 = no visible mycelium, 1 = sparse/dying mycelium, 2 = patchy growth with signs of decline, 3 = healthy mycelium covering but with clear visible substrate, 4 = dense, vigorous mycelium some substrate visible, and 5 = dense mycelium mat without any visible material below the mycelium. Bacterial concentration changes were expressed as log_10_(CFU/g + 1) differences between 14 dpi and 0 dpi to allow for inclusion of samples below the detection limit. A +1 offset was applied to permit log-transformation of zero counts, and the number of below-detection-limit samples per species is reported with the results. Contamination was classified as binary (presence/absence) and included as a fixed effect in species × contamination interaction models to assess whether contamination modified species effects on physiological parameters. For visualization, contamination was further categorized by area (<20 cm²/≥20 cm²). The null hypothesis for all models was that fungal species had no effect on the response variable (growth area, bacterial concentration change, pH, or dry weight loss). Linear mixed-effects models were fitted using the lmer function of the lme4 package. Residual diagnostics showed unequal residual variance among treatments, driven by the uninoculated controls, which split into mold-free acidic plates and mold-colonized alkaline plates. Models were therefore refitted with treatment-specific residual variances for all responses except dry weight. The function lme of the package was used nlme for models with treatment-specific residual variances. The *P*-values for fixed effects were obtained via Satterthwaite’s degrees of freedom approximation (lmerTest) for lme4 fits and via nlme’s default containment degrees of freedom for the treatment-specific-variance fits. Pairwise comparisons were adjusted within each model using Tukey HSD but not across response variables. Species × contamination interaction contrasts were adjusted using the false discovery rate rather than Tukey HSD; because several contamination-by-species cells contained few or no plates, these interaction results are interpreted descriptively rather than as confirmatory tests. The area under the curve (AUC) was calculated using all available measurement timepoints per treatment via the trapezoidal rule, independent of timepoint exclusions applied for trajectory visualization. All values are reported as mean ± SE unless otherwise stated. Growth rate was calculated as the mean area expansion rate (cm²/day), defined as colony area at the species-specific reference timepoint divided by the number of days post-inoculation. Reference days were chosen from timepoints imaged in every batch: 2 dpi for *S. fimicola* F35 and 8 dpi (the peak, before dieback) for *T. harzianum* T22, and 14 dpi for the remaining species. The growth trajectories in Fig. 3 were plotted only from the days imaged in every batch. In Fig. 1, 5-dpi photographs were used to represent the early time point, and 14-dpi photographs were used for the endpoint.

**Figure 1:**
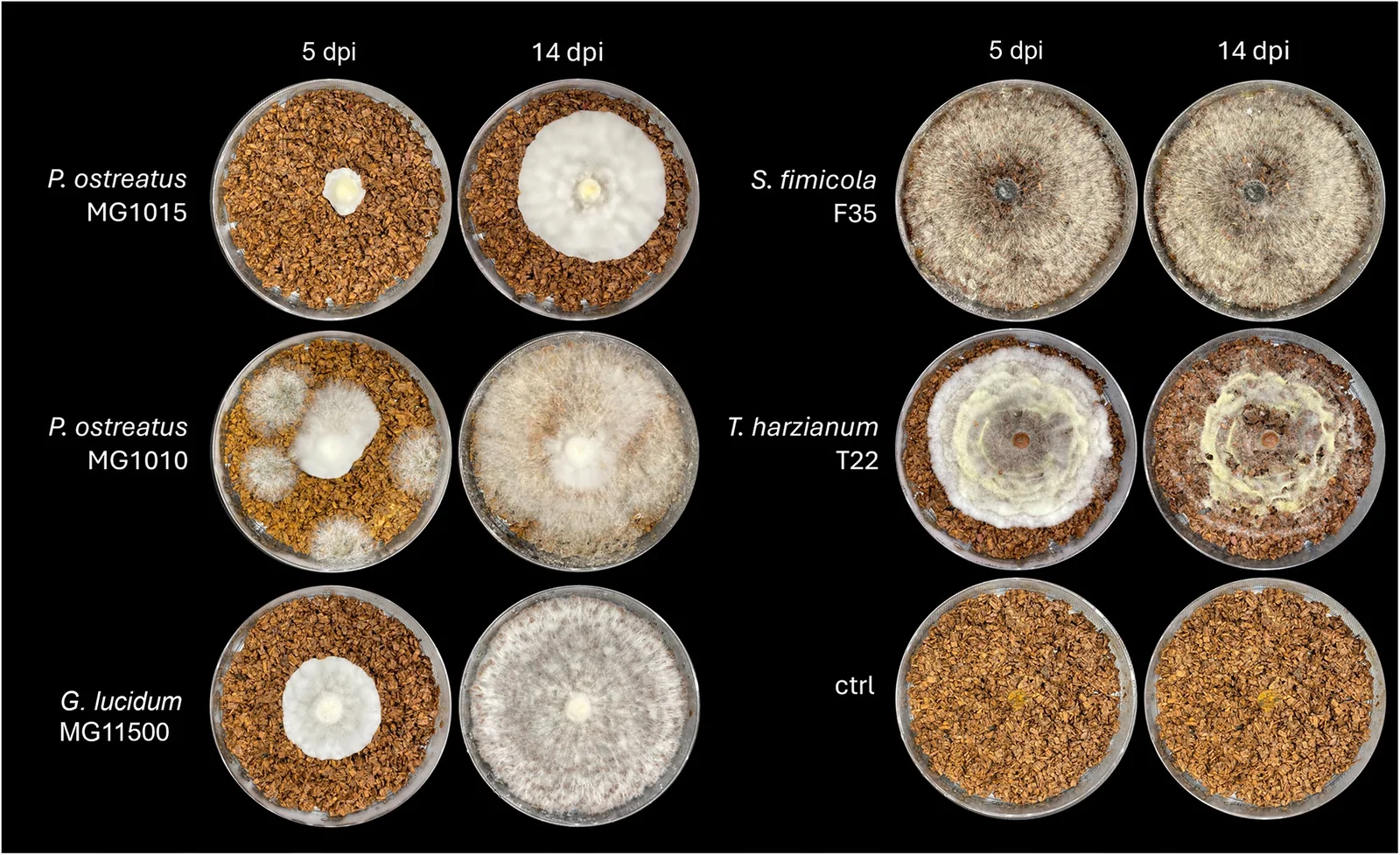
Representative photographs of fungal colonization on fecal-woodchip substrates at 5 and 14 days post-inoculation (dpi). Each panel shows a single replicate plate for the five inoculated isolates and an uninoculated control.

### Use of generative AI

Generative AI was used to produce the code for data analysis, grammatical correction in the text, and reviewing the draft (Claude, Anthropic). The authors take full responsibility for all AI-assisted content.

## Results

### Fungal growth kinetics and colonization success

Distinct patterns of fungal colonization success and contamination across species and fecal samples were observed (Table 1).

**Table 1:** Fungal growth success and contamination incidence across species and fecal samples.

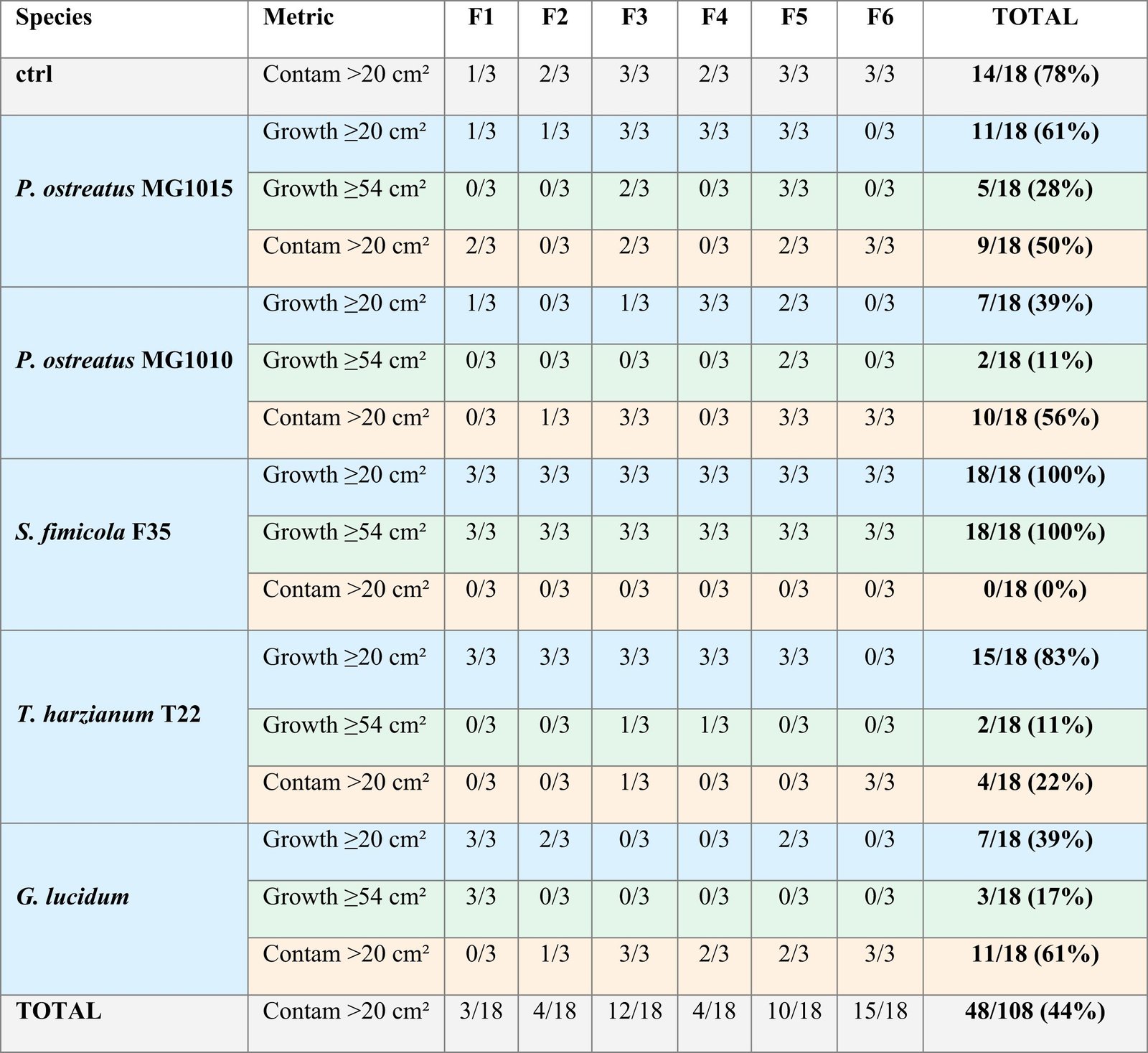

For each species, three metrics are reported: Growth ≥20 cm² (proportion of replicates exceeding the initial growth threshold, ~30% plate coverage); Growth ≥54 cm² (proportion achieving near-complete colonization, ~85% plate coverage); and contamination >20 cm² (proportion of plates with spontaneous mold contaminants exceeding 20 cm²). Values are presented as count/total replicates per fecal sample (columns F1–F6) and as count/total (percentage) in the TOTAL column. Control treatments (ctrl) are shown for contamination only. Row colors distinguish metrics: blue = Growth ≥ 20 cm², green = Growth ≥ 54 cm², and orange = Contamination > 20 cm².

Growth success (≥20 cm², ~30% plate area) was set as a threshold for retaining a sample for further analysis, with sufficient area to sample from within the mycelium growth zone. The second threshold (≥54 cm², ~85% plate coverage) was set to show if the fungus grew fast enough to outcompete spontaneously appearing molds. The number of replicates reaching this first threshold varied considerably among species. *S. fimicola* F35 achieved the highest overall success rate, with consistent growth across all fecal samples. *Trichoderma harzianum* T22 similarly demonstrated robust colonization capacity but failed to reach this in fecal sample 6 with high contamination. In contrast, *G. lucidum* showed the lowest growth success, with notable variation between fecal samples, indicating great sensitivity to substrate conditions. At the ≥54 cm² threshold, *S. fimicola* F35 reached a 100% success rate, whereas the other fungi exhibited lower rates: *P. ostreatus* MG1015 (28%) and MG1010 (11%), *G. lucidum* (17%), and *T. harzianum* T22 (11%) (Table 1). The difference between the growth ≥20 cm² and ≥54 cm² metrics highlights that while many strains were able to initiate growth, only few managed complete substrate colonization and stay dominant until 14 dpi. This result was either due to slow growth in, e.g. *P. ostreatus*, or because of being blocked by contaminants, or dieback, in the case of *T. harzianum* (Figs 1 and 2).

**Figure 2:**
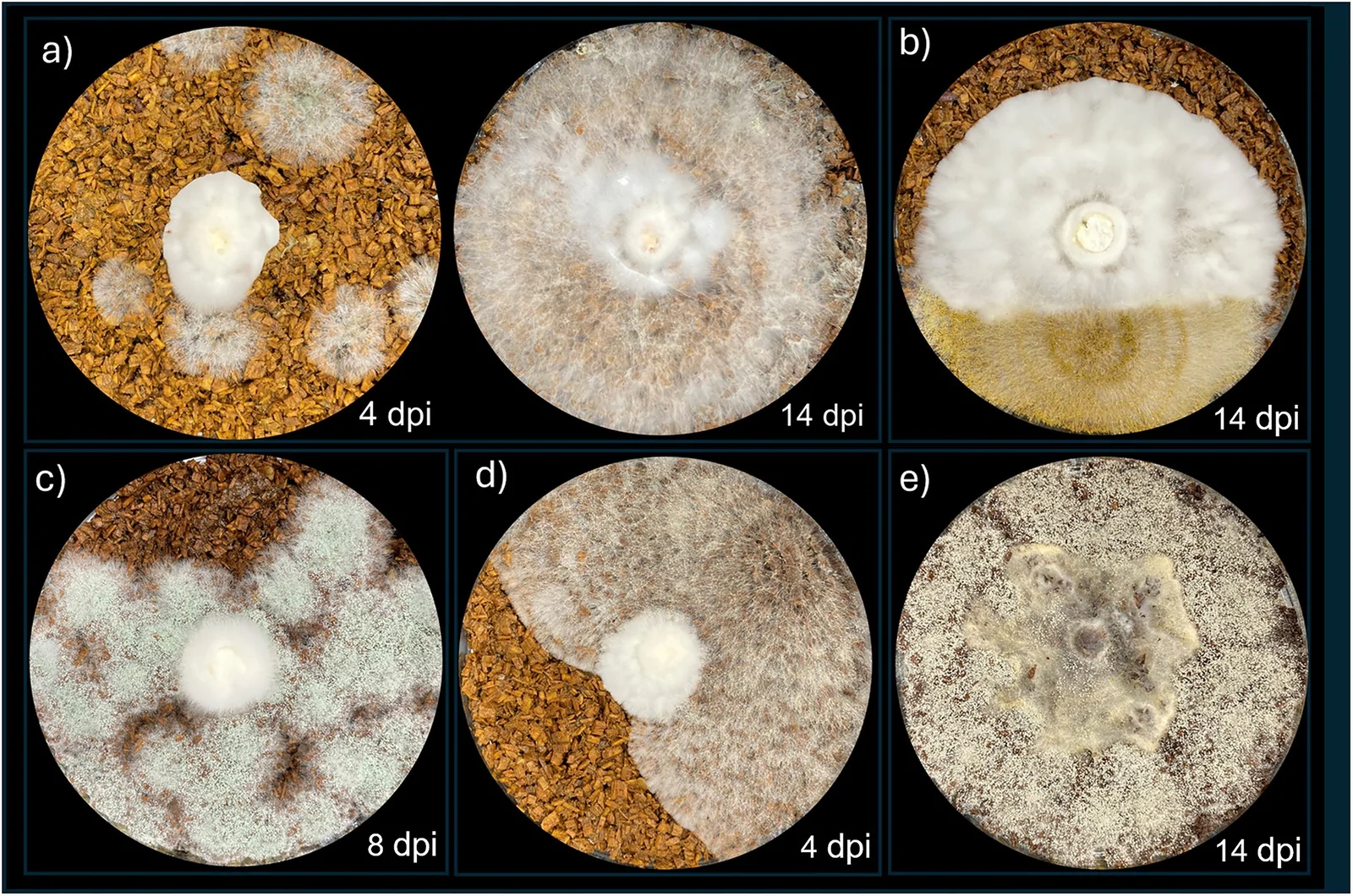
Examples of fungal-contaminant interactions observed on fecal-woodchip substrates. (a) *Pleurotus ostreatus* MG1015 overgrowing a mold colony; (b) *P. ostreatus* MG1015 with a strict contact line where mold blocked further expansion; (c) *P. ostreatus* MG1015 coexisting with mold colonies that block further expansion; (d) mold encircling *G. lucidum* MG11500, blocking radial expansion; and (e) spontaneous mold blocked *T. harzianum* T22 from further spreading.

Contamination incidence showed important patterns. Control treatments exhibited baseline contamination levels, representing the inherent fungal spore load of fecal substrates without competition. Among inoculated treatments, *S. fimicola* F35 showed the lowest contamination rates with no contaminations reaching the area size of ≥20 cm^2^. *Trichoderma harzianum* was the second fastest growing fungus and achieved the second-best mold suppression, suggesting rapid colonization is vital for effective competitive exclusion of contaminants. Both *P. ostreatus* strains, MG1015 (50%) and MG1010 (56%), and *G. lucidum* (61%) displayed higher contamination rates, related to slower growth rates, allowing opportunistic microorganisms to germinate and establish.

Between-sample variability was evident across all metrics, with some fecal samples showing consistently higher contamination regardless of treatment. This result justifies the use of mixed-effects models with fecal samples as a random effect in subsequent analyses. For all following analyses, only plates with active fungal growth >20 cm^2^ at 14 dpi are used.

*Sordaria fimicola* had the significantly highest growth rate (17.53 ± 0.73 cm²/day). It expanded rapidly, covering roughly 35 cm² of the plate by 2 dpi; in the batches where 3 dpi was recorded, colony area had already reached near-complete plate coverage (∼59 cm²) by that day, after which growth plateaued through 14 dpi (Fig. 3). Further growth was limited by plate size with mycelium looking healthy and dense at 14 dpi (Figs 2 and 3). *Trichoderma harzianum* T22 grew fast with a growth rate of 6.32 ± 0.08 cm²/day with small variation; a short establishment phase of 2 days with slower growth was visible. After 8 dpi, the area started decreasing due to dieback of the mycelium (Figs 2 and 3). *Peurotus ostreatus*, MG1015 and MG1010, and *G. lucidum* had similar growth rates 3.5 ± 0.27 cm²/day, 3.17 ± 0.34 cm²/day, and 3.6 ± 0.3 cm²/day, respectively, with higher variations compared to the other two (Fig. 3). They also had a longer phase with low area expansion until 5 dpi. *Sordaria fimicola* F35 had a significantly higher AUC value (706.2 ± 8.1) compared to *T. harzianum* (462.8 ± 11.9), *P. ostreatu*, MG1015 (230.7 ± 27.8) and MG1010 (215.6 ± 40.0), and *G. lucidum* (241.8 ± 19.0) over the 14-day experiment (Fig. 3).

**Figure 3:**
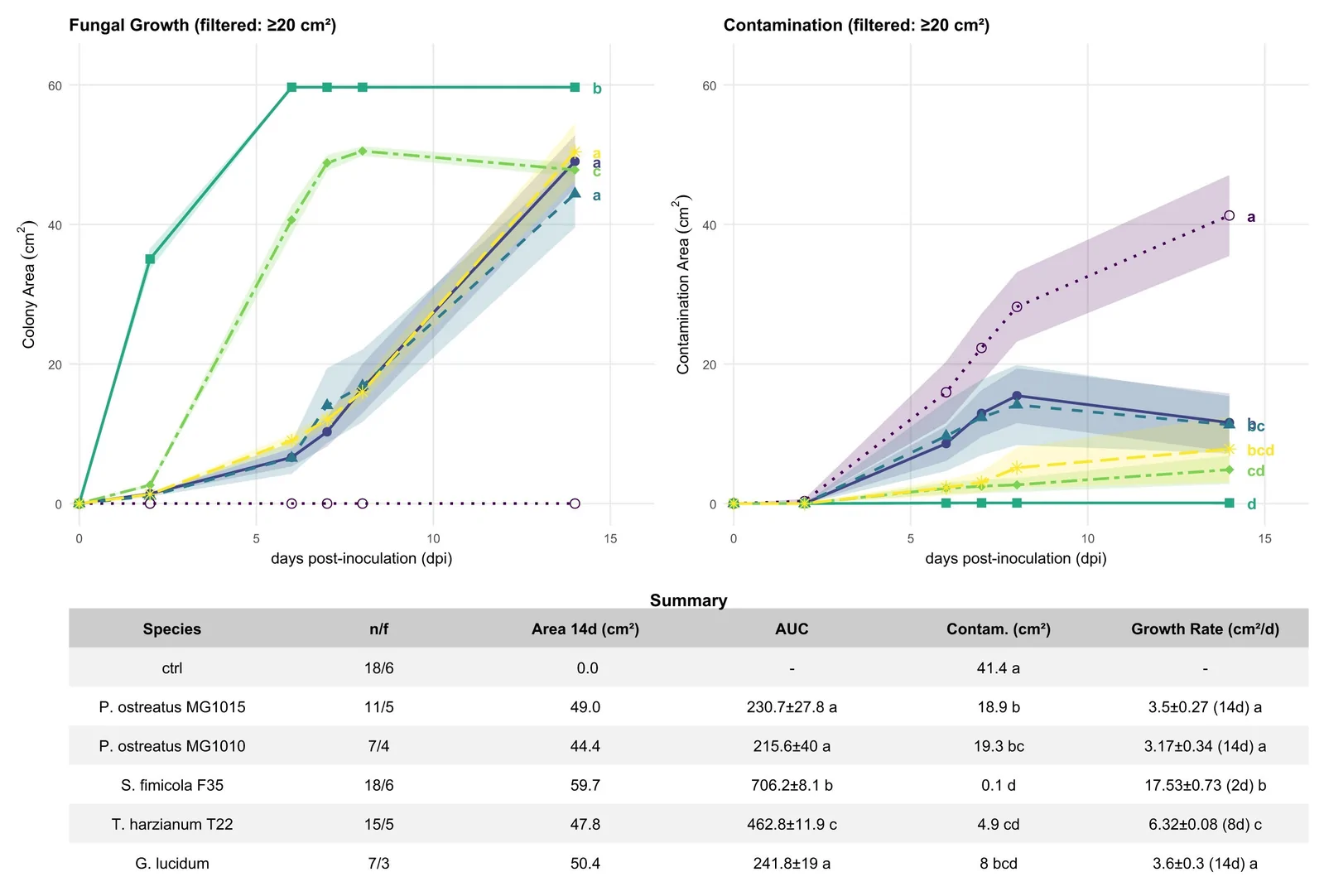
Fungal growth and contamination trajectories over 14 days post-inoculation (dpi) for treatments with successful colonization (≥20 cm² at 14 dpi). (Left) Mean colony area (cm²) for each species; shaded ribbons represent ± SE and lines connect mean values at each measurement timepoint. (Right) Mean spontaneous contaminant area (cm²) over the same period, plotted in the same way. Series are distinguished by color, line style, and point symbol: uninoculated control (dotted line, and open circles); *P. ostreatus* MG1015 (solid line, and filled circles); *P. ostreatus* MG1010 (dashed line, and filled triangles); *S. fimicola* F35 (solid line, and filled squares); *T. harzianum* T22 (two-dash line, and filled diamonds); *G. lucidum* (long-dash line, and asterisks). (Bottom) Summary table reporting sample sizes as *n* (treatments)/*f* (fecal samples), mean colony area at 14 dpi (cm²), AUC, mean contamination area (cm²), and growth rate (cm²/day, with the reference dpi in parentheses). Compact letters indicate significant differences among species from linear mixed-effects models with fecal sample as a random effect (Tukey HSD, *P* < 0.05).

### Interactions with contaminants

Contaminant pressure influenced growth outcomes. Fungal contaminations started to appear in waves at 3 and 5 dpi representing the time it took the fungal spores to germinate and for mycelium to become visible on the pictures (Fig. 3). Expansion happened quickly after the initial appearance and slowed down later due to complete plate coverage or being blocked by the inoculated fungi. Seventy-eight percent of the control fecal samples had contaminants larger than 20 cm². Fungal inoculation was able to reduce this ratio in *P. ostreatus* MG1015 to 50%; in MG1010 to 56%; in *T. harzianum* T22 to 22%; in *G. lucidum* to 61%, and in *S. fimicola* to 0% (Table 1). Contaminant appearance varied between fecal samples (Table 1). *Sordaria fimicola* was the only fungus not influenced by mold pressure, always reaching >90% plate coverage. *Trichoderma harzianum* managed to reduce mold contamination in most samples but not in sample 6 with high mold pressure, where the mold outcompeted *T. harzianum*. The slower growth of *P. ostreatus* and *G. lucidum* allowed contaminants to establish on the plates, but different interactions depending on mold species were observed. Some molds were overgrown by the inoculated fungus, while others blocked its further spread creating a strict contact line. Others encircled the slow growing fungus completely (Fig. 2) blocking further spread. Some molds that were overgrown seemed to trigger a faster growth of the inoculated fungi especially in the *P. ostreatus* strains. Where the mycelium overgrew the molds, it exhibited reduced density and visible substrate exposure; conversely, in the absence of mold or at strict contact lines, the mycelium covered the substrate completely (Figs 1 and 2).

### Effect on substrate pH, dry weight, and fecal indicator bacteria concentrations

To evaluate the effect of the fungal mycelium, substrate samples were collected from initial fecal samples at 0 dpi and from colonized zones at 14 dpi and analyzed for *E. coli* and *Enterococcus* concentrations, dry weight, and pH. Because these analyses included only plates exceeding the 20 cm² colonization threshold, the physiological results represent a best-case scenario among successfully colonized plates, particularly for species with lower colonization success.

#### Bacterial concentrations (*E. coli* and *Enterococcus*)

In the absence of fungal growth, control plates showed a massive decline in *E. coli* concentrations (−6.34 ± 0.41 log, *n* = 4). Conversely, spontaneous mold growth in controls correlated with an *E. coli* increase (+0.81 ± 0.88 log, *n* = 14). *Sordaria fimicola* F35 and *T. harzianum* T22 increased *E. coli* (3.02 ± 0.31 log and 2.94 ± 0.31 log) and *Enterococcus* (1.1 ± 0.46 log and 1.35 ± 0.46 log), respectively. The two *P. ostreatus* strains showed different levels of reduction: MG1010 achieved moderate suppression (−2.02 ± 1.31 log *E. coli*; −2.18 ± 1.14 log *Enterococcus*), while MG1015 resulted in weaker reductions of − 0.57 ± 0.38 log and − 0.43 ± 0.45 log. Among successfully colonized plates (≥20 cm²), *G. lucidum* demonstrated the strongest antibacterial activity, reducing *E. coli* (−5.11 ± 1.36 log) and *Enterococcus* (−5.42 ± 1.12 log) with concentrations lower than the detection limit in most samples (5 out of 7 for *E. coli* and 6 out of 7 for *Enterococcus*). Strong mold presence increased *E. coli* and *Enterococcus* concentrations in *G. lucidum* treated plates (Fig. 4a).

**Figure 4:**
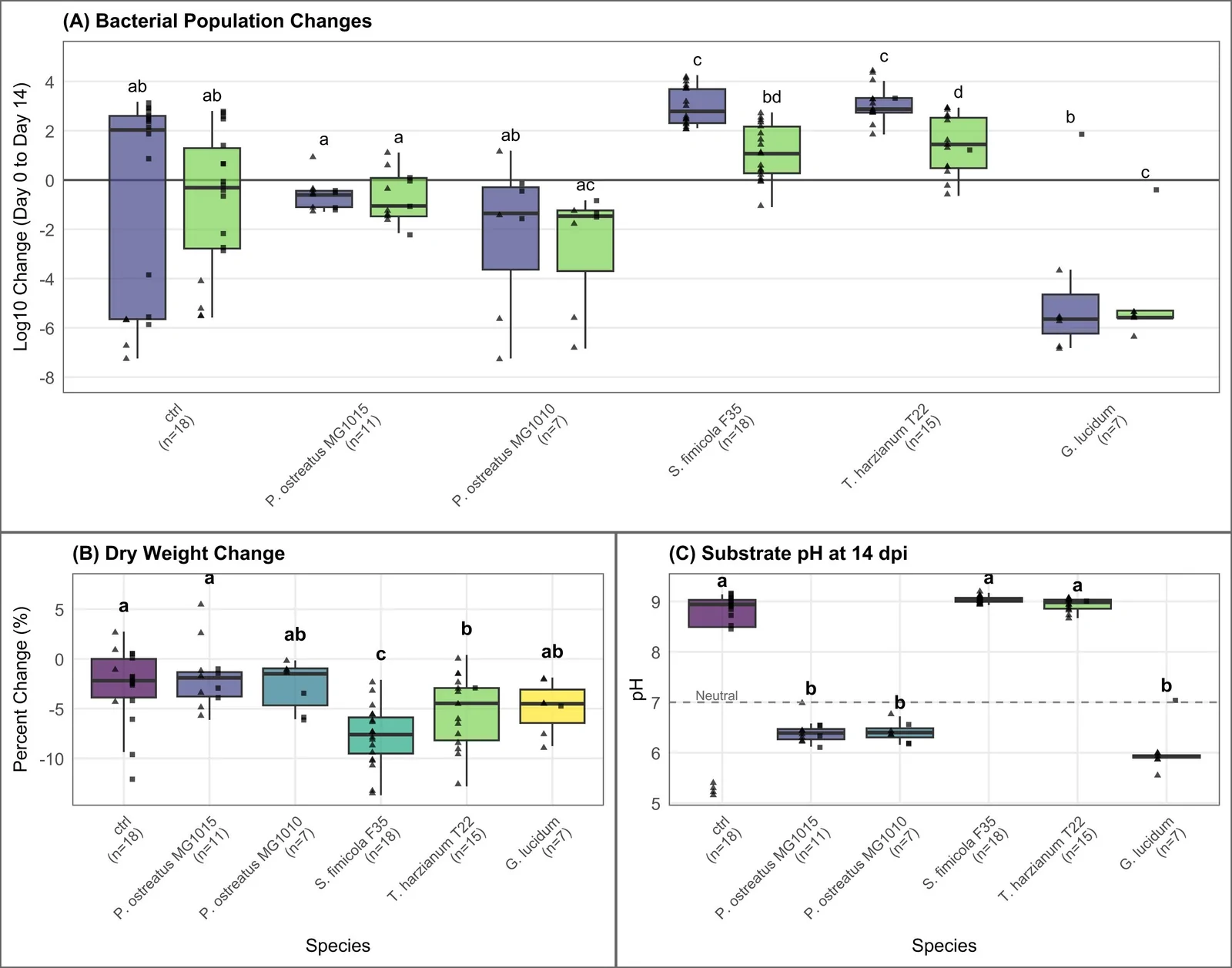
Physiological effects of fungal colonization. (a) Log10 changes in bacterial concentrations from day 0 to day 14. (b) Percent dry weight change. (c) Substrate pH at 14 dpi. In panel a, box fill color denotes the bacterium: *E. coli* (purple) and *Enterococcus* (green). In all panels, point symbols denote the spontaneous-contamination level of each plate: triangles = low (<20 cm²) and squares = high (≥20 cm²); in panels b and c, the box fill distinguishes species, which are also labeled on the horizontal axis. Letters above boxes indicate significant differences from linear mixed-effects models with Tukey-adjusted pairwise comparisons (*P* < 0.05); groups sharing a letter do not differ significantly.

#### Dry weight change

Substrate dry weight changes varied significantly among species. The mixed-effects model showed significant species effects (*P* < 0.05) with an intraclass correlation coefficient of 0.68. *Sordaria fimicola* F35 and *T. harzianum* T22 showed a dry weight loss with −7.8% ± 1.3% and −5.6% ± 1.3% both being significantly lower than control treatments (−2.6% ± 1.3%). The two *P. ostreatus* strains (MG1010: −4.8% ± 1.4%; MG1015: −2.7% ± 1.3%) and *G. lucidum* (−4.1% ± 1.4%) were not significantly different from the control (Fig. 4b).

In control treatments, contaminated samples (*n* = 14) showed greater weight loss (−3.2%) than uncontaminated samples (*n* = 4; −0.6%). *Ganoderma lucidum* contamination had little effect on the weight loss: −4.8% with contamination compared to −4.9% without.

#### Fungal influence on pH

The mean pH of the control was 8.11 ± 0.37 but was highly dependent on contaminations. Samples without mold contamination were acidic with a pH of 5.26 ± 0.05, *n* = 4, while those with spontaneous mold became alkaline pH 8.92 ± 0.05, *n* = 14. Similarly, *S. fimicola* F35 (9.03 ± 0.03) and *T. harzianum* T22 (8.96 ± 0.04) resulted in strong alkalization, comparable to the contaminated controls. In contrast, both *P. ostreatus* strains (MG1010: 6.41 ± 0.09; MG1015: 6.41 ± 0.08) and *G. lucidum* (6.07 ± 0.17) maintained an acidic environment (Fig. 4c).

#### Substrate change observation and odor suppression changes

Some fungi had remarkable effects on the substrate. Both *P. ostreatus* and *G. lucidum* suppressed fecal odor and produced a clean, strong mushroom scent. Conversely, plates treated with *S. fimicola* and *T. harzianum* T22 retained a pungent ammonia odor comparable to control treatments. Furthermore, *P. ostreatus* changed the substrate color from brown to straw-colored (Fig. 5).

**Figure 5:**
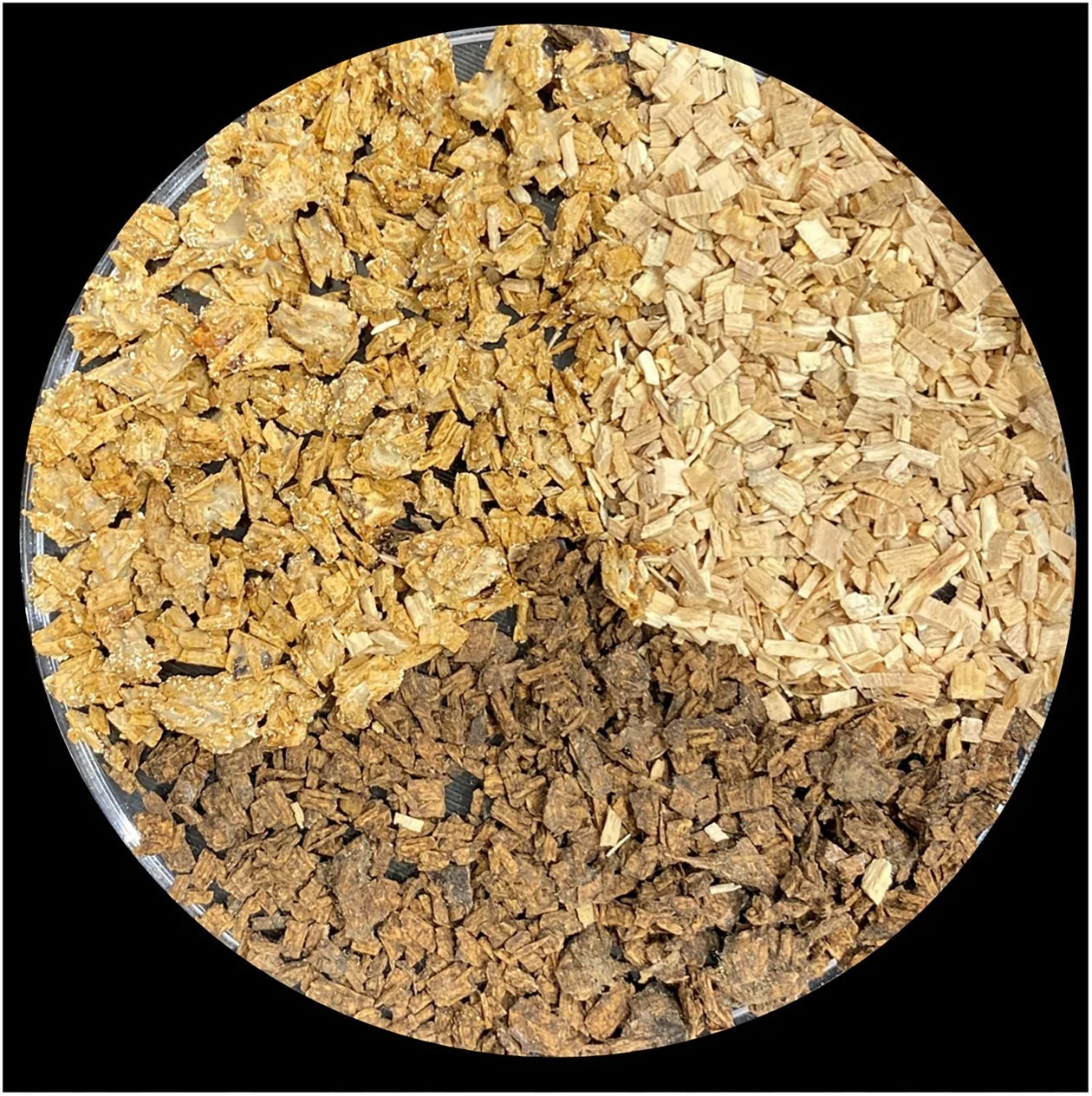
Substrate modifications by fungal colonization. *Pleurotus ostreatus*-treated substrate (top left) at 14 dpi showing color change from brown to straw-colored, compared with control substrate (bottom) and untreated wood chips (top right).

## Discussion

### Overview

To our knowledge, this is the first study to systematically quantify the capacity of different fungi to colonize fresh human feces and to measure the influence of mycelium on pH, fecal indicator bacteria, and dry weight loss. While previous research has explored the fungal influence on composting, mostly in larger composting batches, our results reveal the direct impact of mycelium of the inoculated fungus on small batches of human feces, transferable to UDDT systems. Different fungal species demonstrate distinct growth patterns and impacts on the substrate, specifically pH modulation, bacterial concentrations, and substrate degradation, warranting validation across diverse fecal sources.

### Role of spontaneous mold growth in uninoculated controls

In control treatments without inoculated fungi, the measured variables changed with appearance of spontaneous mold growth. Plates without growing molds resulted in acidic pH, low concentrations of *E. coli* and *Enterococcus* and low dry weight loss. One plausible explanation for these results is the initial formation of short chain fatty acids (SCFAs) produced by bacteria from remaining sugars thus lowering the substrate pH. These SCFAs are known to be toxic for *E. coli* and *Enterococcus* especially in acidic environments (Royce *et al*. 2013, Zhan *et al*. 2022). This suppressive effect likely resulted in plates where general metabolism came to a halt also resulting in low dry weight loss in these control plates. In a real toilet system with aerial mold spores and multiple additions of fecal material and additives, mold growth would be inevitable. If spontaneous molds started to grow, then the pH would likely increase to >8 as ammonia formation shifts the pH to alkaline (Suzuki 2009). The growing molds degraded the SCFAs resulting in a deprotonation of the ammonium (NH_4_^+^) to ammonia (NH_3_) increasing the pH (Emerson *et al*. 1975). Additionally, fungal-secreted proteases could accelerate protein degradation to further increase ammonia concentrations. We hypothesize that the removal of the suppressive SCFAs by fungal metabolism may explain coliform regrowth in plates with fungal additions. Additional mechanisms that could contribute include increased nutrient availability through fungal enzyme secretion and the breakdown of complex molecules, as well as mycelial networks serving as physical scaffolds (“fungal highways”) along which bacteria could spread, with mycelium potentially creating favorable microhabitats and providing exudates and water on its surface (Kohlmeier *et al*. 2005). However, spontaneous mold growth can lead to high spore exposure posing a risk for mycosis, especially for immunocompromised patients (O’Gorman 2011, Alqarihi *et al*. 2023, Apostolopoulou et al. 2026). Reducing spore exposure in the toilet environment is therefore desirable.

### Species-specific effects of fungal inoculation

The inoculated fungi showed species-specific growth patterns, mold suppression, effects on pH as well as differently influenced bacteria concentrations. *Sordaria fimicola* demonstrated superior growth potential, occupying most of the surface area by 3 dpi. It rapidly and reliably colonized and suppressed molds across all tested fecal samples. This result aligns with its ecological role as an early dung colonizer, where it is one of the first fungi fruiting on herbivore dung (Richardson and Watling 1968). *Sordaria fimicola* also caused the highest dry weight reduction in the experiment. However, together with *T. harzianum* it produced the largest increases in *E. coli* and *Enterococcus* concentrations in the experiment and increased the pH to 9. Research looking at *S. fimicola* and bacteria interaction is scarce, but our experiment showed that both fecal indicator bacteria strongly profited from its presence, even more than from spontaneous mold growth. In contrast, *G. lucidum* had a strong suppressive effect on the bacteria concentration; in most cases below the detection limit. The two *P. ostreatus* strains had a moderately reducing or stabilizing effect on both bacteria but not substantively reducing it as much as *G. lucidum*. Fungal extracts of both species have shown broad antibacterial activity, with the effect of *G. lucidum* being stronger on *E. coli* (Younis *et al*. 2015, Cör Andrejč *et al*. 2022).

Notably, uninoculated control plates without spontaneous mold growth also showed substantial bacterial decline under acidic conditions, raising the possibility that the antibacterial effect observed in *G. lucidum* and *P. ostreatus* treatments may be primarily pH-mediated rather than due to active antimicrobial compound secretion. Separating pH-driven bacterial suppression from direct fungal antimicrobial activity would require pH-buffered controls, which were not included in this study. Furthermore, *G. lucidum* and *P. ostreatus* were able to keep the pH value of the substrate acidic until the end of the assay, keeping it in their optimal growth zone probably by producing organic acids (Tsao 1963, Jayasinghe *et al*. 2008). Keeping the substrate acidic throughout the degradation process could lead to lower ammonia volatilization thus reducing nitrogen loss to the air and in turn, suppressing the smell (Qasim *et al*. 2017). While having beneficial influences on the substrate pH and fecal indicator bacteria concentrations, neither fungus reliably grew on the fecal material. Initial growth was slow and delayed, allowing molds to grow, and sometimes the fungus even failed to start growing. This resulted in low success rates under the conditions tested. However, the small agar plug of inoculum (5 mm diameter) used in this screening delivered a relatively low fungal biomass; the slow establishment of these species may partly reflect an insufficient inoculum dose rather than intrinsic unsuitability. Grain spawn or colonized woodchip inoculum at substantially higher inoculum loads could improve establishment. Further adaptation of the substrate composition and inoculation method would be necessary to evaluate their true potential.

*Trichoderma harzianum* T22 started as the second fastest fungus in the assay, colonizing a large part of the plate until 8 dpi when it started to die back. After dieback, it was overgrown by molds. Unlike the two basidiomycetes, it could not control the substrate’s pH even though it has been proven to manipulate medium pH to its growth optimum in other experiments (Steffen *et al*. 2026). The high pH together with high ammonia concentrations outside its growth range, likely caused its dieback (Onilude and Seyi-Amole 2018).

Several other *Trichoderma* species have been tested on similar substrates and pure fecal material resulting in comparable results (Jules Peter, unpublished data). Also, *Trichoderma* spores could still be a problem for air quality in the toilet environment. *Trichoderma* therefore is not considered to be suitable for application in dry toilet substrates. Regarding dry weight loss, the most successful species was *S. fimicola* followed by *T. harzianum* both being significantly different to the control. The other three fungi did not significantly change dry weight loss compared to the control treatment. This observation may be strongly linked to slower growth rates, which limit the time available for material degradation, and the suppression of bacterial concentrations that reduces overall metabolism in contrast to *S. fimicola* and *T. harzianum* where bacterial concentrations were increased. The addition of easier degradable lignocellulose would likely increase dry weight loss in all treatments in this short-term assay. Still, *S. fimicola* and *T. harzianum* produced greater weight loss than the control, both without and with spontaneous mold growth. In our assay *P. ostreatus* suppressed fecal smell as reported by Urieli (2016) and Dahmen *et al*. (n.d.) but we noticed that this is also true for *G. lucidum*. The reduction of smell can be explained by the acidic pH reducing ammonia volatilization as well as the degradation of strong-smelling amines like putrescine (Yamada *et al*. 1965). Degradation of stercobilin (responsible for the brown fecal color) by *P. ostreatus* can explain the noticeable color change (Pakhadnia *et al*. 2009). With this assay, we could show that fungi, if inoculated or spontaneous, are an important factor in degradation of lignocellulose fecal mixtures increasing dry weight loss, modulation of pH, and a suppression or proliferative effect on bacteria present in feces. Longer experimental runs would be necessary to better understand the degradation process of the fecal material by the fungus and how they influence bacteria concentrations over a longer storage time. *Sordaria fimicola*, being naturally adapted to herbivore dung, demonstrated the highest colonization potential; this suggests that other fungi within this niche warrant further testing, as they appear to offer the potential for reliable colonization of human fecal material.

## Conclusion

This exploratory study provides the first systematic quantification of fungal colonization potential on mixed human fecal material, investigating fungi as a treatment strategy rather than just a composting additive. Species-specific effects were observed on fecal indicator bacteria concentrations, pH, and dry weight loss, though these findings are preliminary and require multi-donor validation. *Sordaria fimicola* proved to be an exceptional colonizer, significantly reducing dry weight and excluding mold contaminants; it also increased fecal indicator bacteria concentrations and the pH value. Among successfully colonized plates, *G. lucidum* demonstrated the strongest potential for bacterial suppression, reducing fecal indicator bacteria below detection limits in most samples, although its low colonization success rate and the potential confounding role of acidic pH in bacterial suppression limit the strength of this conclusion. *Pleurotus ostreatus* had a moderately reducing or stabilizing effect on fecal indicator bacteria but kept the substrate pH acidic and led to a distinct color change in fecal material, removing the fecal appearance. Both *G. lucidum* and *P. ostreatus* suppressed fecal odor in qualitative assessments. Yet, both species were limited by slow initial growth and poor competitiveness against molds, though this may partly reflect the small agar plug inoculum used rather than intrinsic unsuitability. *Trichoderma harzianum* was poorly adapted to the substrate and experienced dieback at 8 dpi.

In conclusion, this study demonstrates that fungal inoculation can suppress spontaneous mold appearance compared to non-inoculated controls and, in the fastest-colonizing species (*S. fimicola* and *T. harzianum*), significantly enhance the substrate degradation rate, with species-specific trade-offs between colonization reliability, fecal indicator bacteria dynamics, and pH control. Testing more fungal species for their effect on human fecal material has the potential to support the development of enhanced treatment strategies. Successful species should be tested in multi-donor studies to assess generalizability across diverse human microbiomes; higher-biomass inoculation methods (grain spawn, colonized woodchip) to improve establishment of slow-growing but beneficial species; extended incubation periods; pH-buffered controls to distinguish pH-mediated from direct antimicrobial suppression; and pilot-scale trials in operational dry toilets. Overall, this study provides an early quantitative foundation for the fungal treatment of human feces, pointing to candidate species and the mechanisms that may underlie their effects, and identifying possible research directions from which a practical sanitation strategy could be developed.

## Data Availability

Data is available on https://doi.org/10.5281/zenodo.22231975. *Pleurotus ostreatus*, MG1015 and MG1010, and *G. lucidum* MG11500 are commercially available from Mycogenetics (Germany). *Trichoderma harzianum* T22 was obtained from Agroscope (Switzerland). *Sordaria fimicola* F35 is available from the authors upon reasonable request.
